# The Voluntary Response Index in Electromyographic Study During Landing Test of the Patients With ACL Deficiency: A New Study Protocol

**DOI:** 10.5812/ircmj.14119

**Published:** 2014-05-05

**Authors:** Amin Norouzi Fashkhami, Abbas Rahimi, Khosro Khademi Kalantari

**Affiliations:** 1 Physiotherapy Department, Shahid Behehshti University of Medical Sciences, Tehran, IR Iran

**Keywords:** Anterior Cruciate Ligament (ACL), Coper, Voluntary Response Index (VRI)

## Abstract

**Background:**

Daily Increased rate of anterior cruciate ligament (ACL) injuries in athletes calls for more investigation in these patients to differentiate copers from noncoper ACL-deficient (ACLD) knees as soon as possible.

**Objectives:**

The current study aims to introduce a new electromyographic protocol, named voluntary response index (VRI), that might help to categorize and differentiate patients with ACLD knee from others in the early stage.

**Materials and Methods:**

Thirty-four patients with ACLD knee were allocated into two equal groups, namely, coper and noncoper groups, based upon their ability to return to sport during the preceding six months. The patients with ACLD knee were asked to perform a jump on a force platform from a 75-cm distance.

**Results:**

The results were compared with 17 matched healthy participants. The electromyographic disposable electrodes were attached to the seven muscles of the lower extremity of the participants before performing the test. The outcome measures were the magnitude and similarity index of the VRI, time to stop, vertical ground reaction force, the displacement of the center of pressure’s (COP) path line length, and the participants’ Tegner, IKDC (International Knee Documentation Committee) as well as KOOS (Knee injury and Osteoarthritis Outcome Score) questionnaires scores. Using the appropriate statistical analysis, the electromyographic and force plate data were compared among the three groups. All efforts went into determining whether an association exists between the findings of each group and the participants’ functional questionnaires scores.

**Conclusions:**

The results of this study would be helpful in objectively differentiating the patients with ACLD knee into coper and noncoper groups to receive appropriate treatments before their return to the competitions.

## 1. Background

Tearing of the anterior cruciate ligament (ACL) is one of the most common musculoskeletal injuries affecting athletes with the frequency rate of 250 000 injuries per year or 30 people in 100 000 population in the United States (1,2). It costs more than two billion dollars per year including diagnostic radiology, reconstructive surgery, bracing, and rehabilitation expenses. Moreover, psychological problems to these subjects, particularly to the professional athletes who should leave competitions between six to nine months, must be added (3). In Australia, only reconstructive surgeries cost 75 million dollars annually (4). Seventy percent of all ACL injuries occur during noncontact injuries, particularly during landing in a jump or shearing forces (5). Unfortunately, although there is a rapid increase in ACL injuries during sport activities in Iran, there is no official record in this area. It seems necessary to achieve a deeper understanding regarding the extent that ACL deficiency affects these athletes and to understand more about the ways these individuals cope with stability threatening conditions. It needs more advanced equipment and techniques to analyze these patients to facilitate reducing its adverse effects (6, 7). Traditionally, individuals with the ACL-deficient (ACLD) knee are divided into coper and noncoper groups based on their ability to return to the preinjury level of function (8-10). For more than 50 years, the kinesiological electromyography (KEMG) has been used regularly in physiotherapy research area to study the functional activities (11). Surface (kinesiological) EMG and force platform are two supportive systems that help researchers in more efficient evaluation of the patients with ACLD knee, particularly in dynamic tests. The parameters normally used in routine EMG are the onset/offset and the intensity (root mean square [RMS]) of the muscular activities. In addition to the routine electromyographic studies of individual muscles responsible in a task, studying all muscles responsible for the entire prototype of a task would be valuable (12-15). In this recently introduced method, the electromyographic activities of some muscles are qualitatively and quantitatively evaluated; this method is supposed to be a more objective assessing measure. This protocol is called Voluntary Response Index (VRI) (12-15). In fact, instead of studying each muscle activity individually, the magnitude and the similarity of activities of all muscles involved in a task is studied as a whole in a work pattern. Two items are studied in this protocol: the magnitude and the similarity index. The magnitude is the spatial summation (the resultant) of the RMS of all muscles involved in that task and is calculated by a specific formula (Formula 1). In this method, instead of calculation of the maximum voluntary contraction (MVC) of each muscle, the magnitude of each testing group is compared with the magnitude of normal healthy subjects as a reference group. The similarity index states the extent that the resultant (prototype) vector of each testing group is similar to the prototype of the reference group. The VRI technique was studied in some special cases of the shoulder joint as well as cervical spine and in patients with partial spinal cord injury (12-15). However, no research has been conducted on knee injuries such as ACLD knees. Some clinical observations show that many ACLD subjects may be able to walk with no pain and discomfort, but they express difficulties during maneuvers need acceleration/deceleration or tasks such as landing and jumping that need higher dynamic stability. Therefore, differentiating coper and noncoper ACLD knee is very important. Landing is routinely used as a difficult maneuver to test the functional abilities of subjects with ACLD knees (16).

In brief, due to the high rate of ACL-deficiency in athletes and shortage of useful information provided by the study of the activities of individual muscles, a comprehensive EMG protocol to study the prototype of all muscles involved in a hard task such as landing, as the commonest cause of ACL-rupture, is necessary.

## 2. Objectives

The current research aimed to introduce the VRI technique to study the magnitude and the similarity index of seven muscles of the lower extremity in both coper and noncoper ACLD subjects and to compare them with the matched healthy participants.

## 3. Materials and Methods

This case-control study assessed the coper and noncoper individuals with ACLD knees and compared their results with that of matched healthy controls. We used a convenience sampling, which is a nonrandom sampling method. The ethical approval code was achieved before conducting the study.

### 3.1. Study Population

Thirty-four recreational athletes with age between 20-29 years old who had ACL-deficiency at least six months ago or longer will be recruited in this study. The subjects were allocated into coper and noncoper groups (17 patients in each group) based upon the idea of Fitzgerald et al. (2000) (16). All subjects had to be referred by one orthopedic surgeon based on the clinical examination and magnetic resonance imaging (MRI) findings. Seventeen healthy individuals who were matched with patients with ACLD knee through age, sex, and the level of activities were also recruited as the control group. The patients were recruited based on the inclusion and exclusion criteria including lasting at least six months (25 weeks) post-injury, no more than three score (out of ten) of pain in VAS score, and no swelling or other associated meniscal or musculoskeletal injuries in their knees.

The subjects were recruited based on the convenience sampling via the following formula:

$$n=2\times {[(z_{1-\frac{a}{2}}+z_{1-\beta })\times \sigma /\mu_{1}-\mu_{2}]}^{2}$$

Using a pilot study, estimation of variance was 0.0015 and means were 0.85 and 0.89, respectively.

### 3.2. Data Collection

All subjects were asked to sign an inform consent regarding their voluntarily participation in the study and to complete the published Farsi version of the KOOS, IKDC, and Tegner activity scale questionnaires (17-19) as standard questionnaires for evaluating functional activities. The main index for allocating the patients into coper and noncoper groups was their ability to return to sports with their pre-injury level.

### 3.3. Electromyography

The EMG activities of the seven muscles of the lower extremity including the vastus medialis, vastus lateralis, biceps femoris, semitendinosus, gastrocnemius (lateral and medial heads), and the gluteus medius were recorded during a landing test. The electrodes were placed based on the SENIAM’s (Surface Electromyography for the Non-Invasive Assessment of Muscles) recommendations (19). The sites of the electrodes place on the skin were cleaned and the electrodes of an 8-channel portable EMG system (Biometrics Data Log, Oxford Ltd, UK) were attached to the skin. A Kistler force platform (Kistler Company, Switzerland), which is synchronized with the EMG system, was also used in this study. Since Hashemi et al. (2011) reported a time between ten to 65 milliseconds as the time of injury following the landing for these patients (20), studying the electromyographic activities of the muscles of the lower limb in this time period would be fundamental. In the current study and for more convenience, the electromyographic activities of the aforementioned seven muscles were recorded during the first 100 milliseconds following the landing. The EMG outcome measures in this study were the magnitude and the similarity indices of the muscles of the lower extremity during landing on the single leg. To work this out, at first, the RMS of each muscle was calculated and called as “response vector” of that muscle. Then, the response vectors of the seven muscles were placed in formula 1 and the “prototype response vector (PRV)” was calculated, which simply showed the resultant of the magnitude of all muscles in that task. The PRV was calculated in the coper, noncoper, and control groups and was compared amongst them. Thereafter, the similarity index (SI) of all three groups was calculated with use of formula 2 and was compared among the study groups.

Formula 1 shows the magnitude of *n* muscles in a task and Formula 2 shows the similarity index (SI) of a group of muscles in a task, in which the RVi shows the RMS of each muscle, and the PRVi is the prototype response vector of each group muscle act in a task.

(1)

$$R_{\mathrm{norm}}=\frac{R_{1}R_{2}R_{3}\ldots R_{n}}{\sqrt{\sum_{i}^{}R_{i}^{2}}}$$

Ri = Response vector (RMS) of each muscle

(2)

$$\mathrm{SI}=\frac{\sum_{i}^{}({\mathrm{RV}}_{i}{\mathrm{PRV}}_{i})}{|\mathrm{RV}|\mathrm{PRV}}$$

At the end, all efforts were made to find out any association between the VRI and the questionnaires’ scores of each group. In addition, the sensitivity and specificity of the VRI method in differentiating individuals with ACLD knee as copers or noncopers was studied.

### 3.4. Methodology of the Test

Before starting the test, all subjects were asked to stand on the ground and jump vertically for three times to record their maximum height jumping. Then, they were asked to stand 75 cm far from the force platform and jump on it on one leg, i, the injured leg in patients with ACLD and the matched leg in healthy controls. The important point on landing was that the trials was accepted only if the subjects jumped so high that their head was located between the two lights placed in 50% to 60% of their maximum jump height. In other words, any jumps lower or higher than these two lines was discarded as it might ruin the ground reaction force data. The subjects had to jump while standing on both legs, but they had to land only on one leg. The outcome measures of this study were the magnitude and similarity indices of the tested muscles (VRI), the time to stop landing on the force platform, the maximum vertical ground reaction force, and the path line length of the center of pressure (COP) of the participants.

### 3.5. Statistical Analysis

A Kolmogorov-Smirnov test was used to assess whether the recorded data were normally distributed. A multiple regression analysis was used to determine any correlation between the independent and dependent measurements. Finally, a one-way ANOVA analysis was used to study the magnitude, similarity index, and the force platform data between study groups.

## 4. Results

The annual reports of the World Health Organization (WHO) routinely express a rapid increase in musculoskeletal injuries, particularly, ACL-deficiency in both recreational and professional athletes. Finding the coper patients with ACLD knee has great importance as they are able to continue exercising without reconstructive surgeries. Currently, the electromyographic tools are essential diagnostic apparatus in kinesiological research by use of the parameters such as magnitude, duration, onset activation time (latency) and recently the voluntary response index (VRI). The latter parameter may help opening new insights in diagnosis and finding a more objective and scientific way to divide the ACLD knee subjects into coper and noncopers. This also might help to be able to change the potentially noncoper ACLD knee subjects into coper ones in near future.

## 5. Discussion

During landing, the knee joint, as a joint between the ankle and hip joints, encounters the most impact forces to enable the subjects to obtain their balance as soon as possible. During landing, a smooth force transmission from the ground to the trunk requires an intact ACL. Therefore, studying the methods for better controlling the knee joint during landing may help the researchers to understand how some patients with ACLD knee are able to overcome this ligament deficiency (copers). Due to the all aforementioned reasons, it might be helpful if the muscles responsible for landing are studied as a whole so that it might help clinicians for a better evaluation, diagnosis, and treatment plans.
